# Elevated levels of serum alpha-2-macroglobulin associate with diabetes status and incident CVD in type 1 diabetes

**DOI:** 10.1016/j.jlr.2025.100741

**Published:** 2025-01-04

**Authors:** Baohai Shao, Janet K. Snell-Bergeon, Ian H. de Boer, W. Sean Davidson, Karin E. Bornfeldt, Jay W. Heinecke

**Affiliations:** 1 Department of Medicine, University of Washington, Seattle, WA; 2 Barbara Davis Center for Diabetes, University of Colorado Anschutz Medical Campus, Aurora, CO; 3 Department of Pathology and Laboratory Medicine, University of Cincinnati, Cincinnati, OH

**Keywords:** alpha-2-macroglobulin, atherosclerosis, case-cohort design, incident CVD, Cox proportional regression, mass spectrometry, parallel reaction monitoring, proteomics

## Abstract

Atherosclerotic CVD is a major cause of death in individuals with type 1 diabetes mellitus (T1DM). However, conventional risk factors do not fully account for the increased risk. This study aimed to investigate whether serum proteins associate with diabetes status and the occurrence of CVD in T1DM. We used isotope dilution-MS/MS to quantify 28 serum proteins in 228 subjects participating in the prospective Coronary Artery Calcification in Type 1 Diabetes study. We used linear regression to analyze the association between serum protein levels and T1DM status using 47 healthy controls and 134 T1DM patients without CVD and Cox proportional hazards regression to assess their prediction for incident CVD by a case-cohort study using a subcohort of 145 T1DM subjects and a total of 47 CVD events. Of the 28 serum proteins studied, five of them—alpha-2-macroglobulin (A2M), apolipoprotein A-IV, apolipoprotein L1, insulin-like growth factor 2, and phospholipid transfer protein—were significantly associated with T1DM status, with A2M being 1.6-fold higher in T1DM. After adjusting for potential confounders, A2M independently predicted incident CVD, with a mean hazard ratio of 3.3 and 95% CI of 1.8–6.1. In our study, A2M showed the largest increase in serum levels when comparing patients with T1DM to control subjects. A2M also predicted incident CVD, suggesting that it could serve as both a marker and possibly a mediator of atherosclerosis in T1DM. These findings emphasize the importance of specific serum proteins in assessing and managing CVD risk in T1DM.

Diabetes is a major public health problem worldwide (1). Both type 1 and type 2 diabetes mellitus (T1DM and T2DM) associate with a markedly increased risk of atherosclerotic CVD (2, 3). Moreover, CVD is the major cause of morbidity and mortality in people with T1DM (4). Because traditional cardiovascular risk factors have less power to predict cardiovascular events in T1DM patients (5), identifying new risk factors might point toward underlying mechanisms that exacerbate CVD in T1DM (6). This is important because cardiovascular complications take decades to develop, whereas T1DM usually appears early in life. Nevertheless, remarkably little attention has been focused on searching for protein markers of CVD risk in T1DM.

Quantitative proteomics is widely used to identify candidate biomarkers in biological matrices (7, 8). MS/MS is a particularly powerful approach to quantitative proteomics because it reproducibly detects proteins and can be implemented in many different laboratories. A major advantage of using plasma and serum for proteomic analyses is the ready availability of samples from clinical and translational studies. Moreover, analysis of whole plasma or serum provides a direct and unbiased approach to identifying protein biomarkers. Although MS/MS can potentially quantify hundreds or thousands of proteins in plasma or serum, the presence of a few very high-abundance proteins in concert with the wide range of protein abundances (>10^12^ orders of magnitude) makes unbiased protein detection and quantification analytically difficult (9).

One strategy to mitigating these problems is to use a targeted approach in concert with isotope-labeled peptides to detect and quantify proteins (10, 11). A powerful approach involves selective reaction monitoring (SRM), an MS/MS method that increases the sensitivity of an analysis by allowing the instrument to focus its duty cycle on a small number of proteins. A recent targeted proteomics approach using SRM indicated 13 circulating proteins as possible biomarkers of cardiovascular damage progression associated with T2DM (12). Parallel reaction monitoring (PRM), a recently developed advance in SRM, further increases sensitivity and precision by monitoring product ions with high mass resolution, as these ions are less likely to be affected by interfering ions. Moreover, PRM is faster because it does not require prior selection of target peptide transitions, thus reducing the time and effort required to optimize the analysis (13, 14). By analyzing serum and using isotope dilution-MS/MS, we previously showed that serum apolipoprotein C-III (APOC3, a protein carried by apolipoprotein B (APOB)-containing lipoproteins and HDL) predicted CVD and was associated with increased odds of coronary artery calcium in CACTI (Coronary Artery Calcification in Type 1 Diabetes) (15, 16), a large prospective study of major cardiovascular events in T1DM.

In the current study, we used isotope dilution-MS/MS with PRM to quantify 28 serum proteins in 47 healthy individuals and 181 T1DM patients from the CACTI study. Of the 28 proteins selected for analysis, 11 were apolipoproteins (17) and 17 were CVD-related proteins identified in previous studies (15, 16, 18, 19, 20, 21, 22, 23, 24, 25, 26). We determined if relative levels of any of those proteins associated with T1DM status and with incident CVD in T1DM patients.

## Materials and Methods

### Study subjects and design

All study subjects were from CACTI, a prospective study of incident CVD in T1DM patients. We first investigated the association between serum proteins and T1DM status, using linear regression analysis and a cross-sectional study with 134 randomly selected subjects with T1DM (without incident CVD) and 47 healthy controls without diabetes or incident CVD from the CACTI study. We next assessed the association of the serum proteins with incident CVD. This analysis used 145 subjects in a randomly selected subcohort of T1DM subjects (including 11 with incident CVD) and all 47 T1DM subjects with incident CVD events. The clinical characteristics of this subcohort have been reported (15, 23, 27). We analyzed serum samples collected at baseline, before any CVD events occurred, using a case-cohort design and Cox proportional hazards regression analysis, as described in previous studies (15, 23). This method allowed us to conduct analyses on a randomly selected subcohort that accurately represented the entire population (28, 29). The study was approved by the Colorado Combined Institutional Review Board. Samples from all 238 subjects were deidentified, and MS/MS analyses were performed in a blinded manner.

### Materials and reagents

Unless otherwise specified, all reagents were obtained from Sigma-Aldrich (St. Louis, MO). Water and acetonitrile for MS analyses were Optima LC/MS grade (Fischer Scientific, Pittsburg, PA). Formic acid was purchased from EMD Millipore (Billerica, MA). The ^15^N-labeled apolipoprotein A-I (^15^N enrichment 99+%) was produced by using a bacterial expression system as described (28).

### Protein selection

To investigate the relationship between serum proteins and T1DM status, and between serum proteins and incident CVD, we selected 28 serum proteins for analysis by targeted proteomics. Among these 28 proteins, 11 are apolipoproteins, which are key players in vascular biology and atherosclerosis, including APOA1, APOB, APOC3, and APOE (apolipoprotein E). The remaining 17 proteins are also linked to CVD, identified in previous research (15, 16, 18, 19, 20, 21, 22, 23, 24, 25, 26). For instance, low levels of PON1 and PON3 have been shown to correlate with existing CVD, as indicated by coronary artery calcification (24), and low levels of PON1, PON3, APOA4 (apolipoprotein A-IV), and LCAT are associated with incident CVD in chronic kidney disease patients (22). We also included proteins altered in end-stage renal disease patients, as many are related to CVD (21). The full names of the 28 proteins are listed in supplemental Table S1.

### Serum protein digestion

Serum proteins were digested as previously reported (15). Digestion was halted by acidifying the reaction mixture, and the samples were dried and stored at −80°C until LC-MS analysis. A detailed digestion method is provided in the supplemental Data.

### LC-ESI-MS/MS analysis of serum proteins by PRM

To quantitatively measure relative levels of the serum proteins, we used targeted proteomics with PRM using a nanoACQUITY UPLC (Waters, Milford, MA) for peptide separation, and the peptides were analyzed with an ultrahigh-resolution accurate mass Orbitrap Fusion Lumos Tribrid Mass Spectrometer (Thermo Fisher Scientific, San Jose, CA) as previously reported (15, 23). A detailed description of the LC-ESI-MS/MS analysis operated in the PRM mode is provided in the supplemental Data.

The peptides for each protein were selected from those detected by shotgun analysis and from our previous studies (21, 24). For quantification, two or more peptides were selected for 24 proteins and one peptide for four proteins (supplemental Table S1). All peptides selected for each protein were unique to that protein with the exception of one peptide for SAA1. See the supplemental Data for detailed peptide selection.

### Quantifying serum proteins with ^15^N-labeled APOA1 as the internal standard

The targeted PRM data were analyzed using Skyline (version 23.1.0.268, Skyline Team from University of Washington, Seattle, WA) (29) and the relative levels of proteins were quantified by using ^15^N-labeled APOA1 as the internal standard as previously reported (22). A detailed approach is provided in the supplemental Data.

### Protein nomenclature

Protein names are based on gene names and were capitalized (Hugo Gene Nomenclature Committee, https://www.genenames.org/about/guidelines/). For example, apolipoprotein A-I is termed APOA1.

### Statistical analysis

The values of clinical characteristics are median and interquartile ranges for continuous covariates and N (%) for categorical covariates. Difference of serum protein levels between healthy controls and T1DM patients, 95% CI, and *P* values were calculated by linear regression, in which the protein level was used as dependent variable, and T1DM status was used as an independent variable. For the association between levels of serum proteins and incident CVD, hazard ratios, 95% CI and *P* values were calculated by Cox proportional hazards models using a case-cohort design and the “PROC PHREG” function in SAS to estimate the regression coefficients and compute the robust variance estimates of Breslow (30). Detailed statistical analysis approaches and the inclusion of interaction terms (31, 32) are provided in supplemental Data. All statistical analyses were performed with SPSS (Windows version 19, Chicago, IL) or SAS OnDemand for Academics. The human studies reported in our article abide by the Declaration of Helsinki principles.

## Results

### Levels of five serum proteins differ between study subjects with and without T1DM independently of changes in lipid levels

Table 1 presents the clinical characteristics of the 47 healthy control subjects and 134 T1DM subjects without CVD events from a randomly selected subcohort of the CACTI study. T1DM subjects and healthy control subjects exhibited similar BMIs, systolic blood pressure (SBP) and diastolic blood pressure (DBP), and C-reactive protein levels, with comparable percentages of females and current smokers. However, T1DM subjects were younger and had slightly higher estimated glomerular filtration rate (eGFR) levels and higher albumin creatinine ratio (ACR). T1DM subjects showed lower total cholesterol, LDL-C, and triglyceride (TG) levels but higher HDL-C levels. More T1DM subjects were treated with angiotensin-converting enzyme inhibitors and antihypertensive medications compared with controls, although statin treatment percentages were similar between groups.

**Table 1:** Clinical characteristics control nondiabetic subjects and T1DM subjects without incident CVD in CACTI

Characteristic	Healthy control subjects	T1DM subjects without CVD	*P*-value
Number of subjects	47	134	
Age (years)	45 (38–50)	35 (29–44)	<0.0001^a^
Gender (female), N (%)	21 (47.7)	73 (54.5)	0.25^b^
DM duration (years)	N/A	22 (16–29)	
SBP (mm Hg)	115 (108–123)	116 (108–127)	0.61^a^
DBP (mm Hg)	79 (73–85)	77 (72–82)	0.16^a^
BMI (kg/m^2^)	25.4 (23–28.2)	25.9 (22.8–28.0)	0.87^a^
Cholesterol (mg/dl)	192 (175–-214)	166 (146–193)	<0.0001^a^
TGs (mg/dl)	107 (86–165)	73 (55–99)	<0.0001^a^
HDL-C (mg/dl)	49 (36–57.7)	54 (46–64)	0.014^a^
LDL-C (mg/dl)	121 (100–140)	95 (77–114)	<0.0001^a^
HbA1c (%)	5.6 (5.3–5.8)	7.4 (6.7–8.3)	<0.0001^a^
C-reactive protein (μg/ml)	1.24 (0.94–1.84)	1.13 (0.85–1.90)	0.82^a^
Current smoker, N (%)	3 (6.4)	14 (10.4)	0.41^b^
eGFR (ml/min/1.73 m^2^)	104 (92–110)	112 (93–123)	0.049^a^
ACR (mg/g)	3.71 (2.92–5.10)	5.90 (4.23–12.3)	<0.0001^a^
Insulin dose (U/kg/d)	0	0.59 (0.48–0.72)	
Medications			
ACE inhibitor, N (%)	2 (4.3)	45 (33.6)	<0.0001^b^
ARB, N (%)	1 (2.1)	6 (4.5)	0.47
Antihypertensive, N (%)	3 (6.4)	53 (39.6)	<0.0001^b^
Statin (HMG-CoA reductase inhibitor), N (%)	3 (6.4)	18 (13.4)	0.19

Entries are median (interquartile range) for continuous covariates and N (%) for categorical covariates.

ACE, angiotensin-converting enzyme; ACR, albumin creatinine ratio; ARB, angiotensin receptor blocker; BMI, body mass index; DBP, diastolic blood pressure; eGFR, estimated glomerular filtration rate; SBP, systolic blood pressure; TG, triglyceride.

a *P* values from Mann-Whitney *U* test for non-normally distributed variables.

b *P* values from a Pearson Chi-square test for categorical variables.

**Fig. 1:**
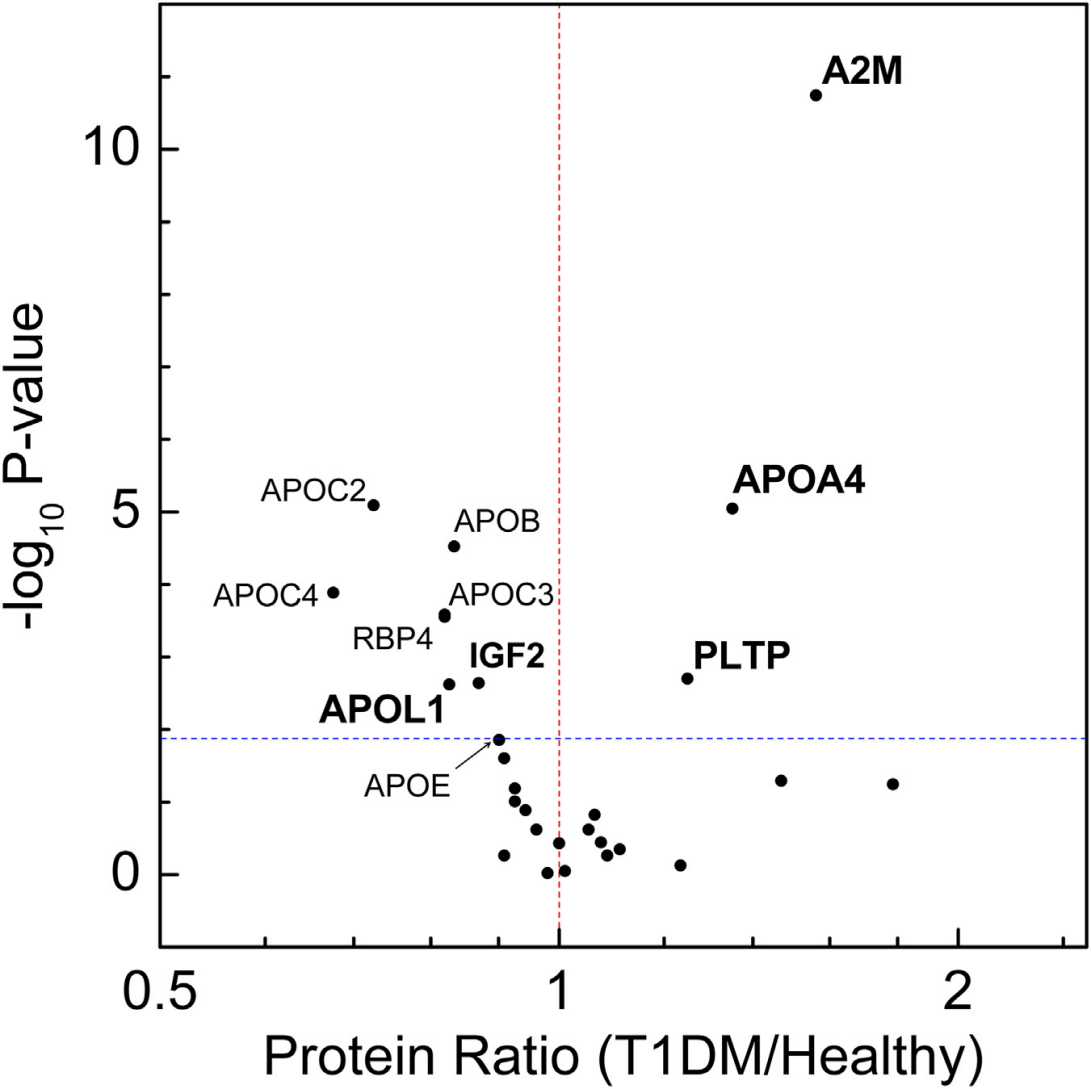
Relative serum protein levels in healthy control subjects and T1DM subjects without CVD. Levels of 28 proteins were measured in serum tryptic digests using isotope dilution-MS/MS with PRM in 134 T1DM subjects without incident CVD and 47 healthy controls. For each protein, the *P* value from the Mann-Whitney nonparametric test is plotted against the log2 fold mean difference in protein levels between T1DM subjects and healthy controls. After adjusting for multiple comparisons, proteins with a *P* value < 0.015 (indicated by the dotted horizontal line) show significant differences in relative abundance. Proteins increased in T1DM subjects have a value >1 on the x-axis (arbitrary units), whereas proteins reduced in T1DM subjects have a value <1. Bolded proteins are significantly associated with T1DM status after controlling for potential confounders. The significant proteins before adjusting for potential confounders include A2M, APOA4, APOB, APOC2, APOC3, APOC4, APOL1, IGF2, PLTP, and RBP4. A2M, alpha-2-macroglobulin; APOA4, apolipoprotein A-IV; APOB, apolipoprotein B; APOC2, apolipoprotein C-II; APOC3, apolipoprotein C-III; APOC4, apolipoprotein C-IV; APOL1, apolipoprotein L1; IGF2, insulin-like growth factor 2; PLTP, phospholipid transfer protein; RBP4, retinol binding protein 4.

We used targeted PRM with ^15^N-labeled apolipoprotein A-I as the internal standard to quantify the 28 serum proteins (11 apolipoproteins and 17 CVD-related proteins, supplemental Table S1). All 28 proteins were detected in every serum sample analyzed. Initially, we compared levels of these proteins between individuals with and without T1DM. After adjusting for multiple comparisons using the Benjamini-Hochberg false discovery rate (5%), PRM analysis revealed that 11 proteins showed differential serum levels between healthy control subjects and T1DM subjects without CVD events. Specifically, the mean levels of three proteins (A2M [alpha-2-macroglobulin], APOA4, and PLTP [phospholipid transfer protein]) were higher, whereas eight proteins (APOB, APOC2 [apolipoprotein C-II], APOC3, apolipoprotein C-IV, APOE, APOL1 [apolipoprotein L1], RBP4 [retinol binding protein 4], and IGF2 [insulin-like growth factor 2]) were lower in individuals with T1DM compared with healthy controls (Fig. 1).

We next conducted unadjusted linear regression analysis to assess the differences in serum protein levels between control subjects and T1DM subjects, providing 95% CI and *P* values. Subsequently, we adjusted these results for multiple comparison using the Benjamini-Hochberg method (5% false discovery rate). This analysis identified 10 serum proteins that significantly associated with T1DM status (supplemental Table S2). Notably, among the 11 proteins initially identified in the T1DM group (Fig. 1), only APOE lost significance after adjusting for multiple comparison. Of these 10 proteins, the levels of three were higher in people with T1DM compared with healthy controls, whereas seven were lower in T1DM (Table 2).

**Table 2:** Five serum proteins significantly associate with T1DM in CACTI

Protein	Level (mean ± SD) (arbitrary unit)		Unadjusted difference (95% CI)	Adjusted difference (95% CI)				*P*-value (model 4)
	Control (N = 47)	T1DM (N = 134)		Model 1	Model 2	Model 3	Model 4	
A2M	0.64 ± 0.20	1.0 ± 0.40	0.36 (0.24, 0.48)	0.25 (0.13, 0.37)	0.23 (0.10, 0.37)	0.22 (0.09, 0.35)	0.25 (0.13, 0.38)	0.00014
APOA4	0.74 ± 0.25	1.0 ± 0.39	0.26 (0.14, 0.38)	0.29 (0.16, 0.42)	0.20 (0.08, 0.33)	0.20 (0.06, 0.33)	0.33 (0.20, 0.46)	<0.0001
APOB	1.21 ± 0.32	1.0 ± 0.36	−0.20 (−0.32, −0.08)	−0.19 (−0.31, −0.06)	−0.14 (−0.26, −0.01)	−0.20 (−0.34, −0.06)	0.02 (−0.07, 0.12)	0.60
APOC2	1.38 ± 0.61	1.0 ± 0.56	−0.38 (−0.57, −0.19)	−0.37 (−0.58, −0.17)	−0.32 (−0.53, −0.10)	−0.43 (−0.65, −0.20)	−0.14 (−0.32, 0.03)	0.11
APOC3	1.23 ± 0.45	1.0 ± 0.56	−0.22 (−0.40, −0.04)	−0.21 (−0.40, −0.01)	−0.21 (−0.41, −0.001)	−0.25 (−0.46, −0.04)	−0.03 (−0.19, 0.14)	0.76
APOC4	1.49 ± 0.96	1.0 ± 0.81	−0.48 (−0.77, −0.20)	−0.51 (−0.81, −0.20)	−0.37 (−0.69, −0.06)	−0.51 (−0.85, −0.18)	−0.12 (−0.37, 0.13)	0.35
APOL1	1.23 ± 0.52	1.0 ± 0.34	−0.21 (−0.35, −0.08)	−0.27 (−0.41, −0.13)	−0.30 (−0.45, −0.16)	−0.31 (−0.47, −0.16)	−0.24 (−0.39, −0.09)	0.0018
IGF2	1.16 ± 0.31	1.0 ± 0.31	−0.15 (−0.25, −0.05)	−0.21 (−0.32, −0.10)	−0.24 (−0.36, −0.13)	−0.25 (−0.37, −0.14)	−0.18 (−0.30, −0.07)	0.0024
PLTP	0.80 ± 0.21	1.0 ± 0.34	0.20 (0.09, 0.30)	0.19 (0.08, 0.31)	0.16 (0.05, 0.28)	0.14 (0.02, 0.26)	0.21 (0.10, 0.32)	0.00035
RBP4	1.23 ± 0.40	1.0 ± 0.46	−0.22 (−0.37, −0.07)	−0.21 (−0.37, −0.06)	−0.31 (−0.46, −0.16)	−0.33 (−0.49, −0.16)	−0.15 (−0.31, 0.01)	0.070

The number of total subjects was 181, including 47 healthy control subjects and 134 T1DM subjects in the subcohort group without CVD events. Protein levels were quantified in tryptic digests of serum by isotope dilution PRM MS/MS. The level of each serum protein from 134 cohort subjects without CVD events was defined as an arbitrary unit of 1.00. Levels are means ± SDs. Difference of serum protein levels between healthy controls and T1DM patients, 95% CI, and *P* values are calculated by linear regression, in which the protein level was used as dependent variable and T1DM status was used as an independent variable. The differences were from the unstandardized coefficients (B). Proteins significantly associated with diabetes status after controlling the Benjamini-Hochberg false discovery rate at 5% are shown. Model 1 is a model adjusted for age and sex (the base model). Model 2 is model 1 further adjusted for nonlipid clinical parameters: SBP, DBP, smoker, eGFR, and log-transformed ACR. Model 3 is model 1 further adjusted for on medications: statin use and antihypertensive medications. Model 4 is model 1 further adjusted for lipid risk factors: HDL-C, LDL-C, and log-transformed TGs. See supplemental Table S1 for protein names.

A2M, alpha-2-macroglobulin; APOA4, apolipoprotein A-IV; APOB, apolipoprotein B; APOC2, apolipoprotein C-II; APOC3, apolipoprotein C-III; APOC4, apolipoprotein C-IV; APOL1, apolipoprotein L1; IGF2, insulin-like growth factor 2; PLTP, phospholipid transfer protein; RBP4, retinol binding protein 4.

We constructed linear regression models that were adjusted for potential confounding variables. Because the analysis included nine parameters that differed significantly between subjects with and without T1DM and some indifferent but important parameters (like sex, smoking status, etc.) (Table 1) but there were only 47 healthy control subjects, we built sequential models to avoid overfitting the data. To obtain a base model, we adjusted our analysis for age and sex. This adjustment did not alter the significance of the associations between the 10 proteins and T1DM status (model 1; Table 2). We next expanded the base model to include potential nonlipid parameters including current smoking status, SBP and DBP, eGFR, and log-transformed ACR. Even with this broader adjustment, the associations between the 10 proteins and T1DM status remained unchanged (model 2; Table 2). We subsequently adjusted the base model for medication use (antihypertensive and statins), but this also failed to modify the associations (model 3; Table 2).

In contrast, when we adjusted the base model for lipid risk factors (HDL-C, LDL-C, and log-transformed TG levels), the associations with T1DM status for five proteins, APOB, APOC2, APOC3, apolipoprotein C-IV, and RBP4, lost significance (model 4, Table 2), strongly suggesting that, for those five proteins, the lower levels of plasma LDL-C and TGs and/or higher HDL-C levels (Table 1) were the primary drivers of the associations with T1DM. In contrast, the other five proteins retained their significant association with T1DM status in all the models adjusted for potential confounders (Table 2).

Levels of three serum proteins—A2M, APOA4, and PLTP—were higher in subjects with T1DM than in control subjects (Fig. 1 and Table 2). Serum levels of A2M exhibited the largest relative difference (>50%) between control subjects and T1DM subjects (0.64 vs. 1.00, healthy vs. T1DM, arbitrary unit). Conversely, levels of two proteins—APOL1 and IGF2—were lower in subjects with T1DM compared with control subjects (Fig. 1 and Table 2). These findings begin to provide insights into the serum proteomic landscape of T1DM.

### Elevated levels of A2M positively associate with incident CVD in T1DM

To assess whether the 28 serum proteins were associated with CVD risk in the CACTI study, we analyzed a subcohort comprising 145 randomly selected T1DM subjects and all 47 cases with T1DM and incident CVD (including 11 within the subcohort) (8, 9). Initially, we performed unadjusted Cox proportional hazards analyses for each protein, followed by adjustment for multiple comparisons using a false discovery rate of 5%. This approach identified 12 proteins significantly associated with incident CVD (supplemental Table S3), including five apolipoproteins: APOA4, APOB, APOC1, APOC2, and APOC3, alongside A2M, AMBP (alpha-1-microglobulin/bikunin precursor), B2M (beta-2-microglobulin), ITIH4 (inter-alpha-trypsin inhibitor heavy chain H4), PLTP, PTGDS (prostaglandin-H2 D-isomerase), and RBP4. These findings highlight potential serum protein markers for CVD risk in T1DM.

As previously reported (8, 9) (Table 3), individuals with CVD were older than T1DM subjects without CVD in the randomly selected subcohort. They also exhibited longer diabetes duration, higher SBP and DBP, elevated hemoglobin A1c (HbA1c), total cholesterol, and TG levels, lower eGFR levels, higher ACR, and higher rates of current smoking. Moreover, the CVD group had a higher prevalence of statin use, angiotensin-converting enzyme inhibitors, and antihypertensive medications. To address these potential confounders, we constructed adjusted Cox proportional hazards models. Given the limited number of CVD events (47 subjects) and 13 significant parameters (Table 3), we employed sequential modeling strategies to prevent overfitting the data.

**Table 3:** Clinical characteristics of T1DM control subjects and T1DM subjects with incident CVD

Characteristic	T1DM control subjects	T1DM subjects with incident CVD	*P*-value
Number of subjects	134	47	
Age (years)	35 (29–44)	45 (38–50)	<0.0001^a^
Gender (female), N (%)	73 (54.5)	21 (44.7)	0.25^b^
DM duration (years)	22 (16–29)	32 (24–37)	<0.0001
SBP (mm Hg)	116 (108–127)	126 (114–136)	0.03^a^
DBP (mm Hg)	77 (72–82)	80 (74–85)	0.02^a^
BMI (kg/m^2^)	25.9 (22.8–28.0)	26.7 (23.5–30.7)	0.24^a^
Cholesterol (mg/dl)	166 (146–193)	183 (163–209)	0.0023^a^
TGs (mg/dl)	73 (55–99)	99 (72–132)	0.00024^a^
HDL-C (mg/dl)	54 (46–64)	55 (41–68)	0.74^a^
LDL-C (mg/dl)	95 (77–114)	97 (84–131)	0.080^a^
HbA1c (%)	7.4 (6.7–8.3)	8.2 (7.6–8.8)	<0.0001^a^
C-reactive protein (μg/ml)	1.13 (0.85–1.90)	1.44 (0.94–2.56)	0.15^a^
Current smoker, N (%)	14 (10.4)	14 (29.8)	0.0016^b^
eGFR (ml/min/1.73 m^2^)	112 (93–123)	93 (61–106)	<0.0001^a^
ACR (mg/g)	5.9 (4.23–12.3)	17.7 (7.07–180)	<0.0001^a^
Insulin dose (U/kg/d)	0.59 (0.48–0.72)	0.60 (0.43–0.73)	0.69^a^
Medications			
ACE inhibitor, N (%)	45 (33.6)	27 (57.4)	0.0040^b^
ARB, N (%)	6 (4.5)	4 (3.0)	0.308^b^
Antihypertensive, N (%)	53 (39.6)	35 (74.5)	<0.0001^b^
Statin (HMG-CoA reductase inhibitor), N (%)	18 (13.4)	24 (51.1)	<0.0001^b^

Controls were subjects without incident CVD in the randomly selected T1DM subcohort, and cases were all subjects with incident CVD in CACTI (previously reported (23)). Values are median value (interquartile range) for continuous covariates and N (%) for categorical covariates.

ACE, angiotensin-converting enzyme; ACR, albumin creatinine ratio; ARB, angiotensin receptor blocker; BMI, body mass index; DBP, diastolic blood pressure; eGFR, estimated glomerular filtration rate; SBP, systolic blood pressure; TG, triglyceride.

a *P* values from Mann-Whitney *U* test for abnormally distributed variables.

b *P* values from a Pearson Chi-square test for categorical variables.

We constructed four models to assess the relationship between serum proteins and incident CVD in individuals with T1DM. In model 1, which served as the base model, we adjusted for established clinical risk factors (age, sex, T1DM duration, and eGFR) and found that eight proteins (A2M, AMBP, APOB, APOC1, APOC2, APOC3, ITIH4, and RBP4) were significantly associated with incident CVD (model 1, Table 4). Model 2 added nonlipid clinical factors (smoking status, SBP, DBP, HbA1c, and ACR) to the base model, and the same eight proteins remained significant (model 2, Table 4). In model 3, after adjusting for medication use (antihypertensives and statins), six proteins (A2M, APOB, APOC1, APOC2, APOC3, and RBP4) continued to show significant associations with incident CVD (model 3, Table 4). In model 4, which included lipid parameters (HDL-C, LDL-C, and log-transformed TGs), only A2M remained significantly associated with incident CVD (model 4, Table 4).

**Table 4:** Serum levels of A2M significantly associated with incident CVD in T1DM subjects

Protein	Levels (mean ± SD) (arbitrary unit)		Unadjusted hazard ratio (95% CI)	Adjusted hazard ratio (95% CI)				*P*-value (model 4)
	T1DM cohort (N = 145)	T1DM CAD cases (N = 47)		Model 1	Model 2	Model 3	Model 4	
A2M	1.01 ± 0.40	1.17 ± 0.45	1.60 (1.17–2.19)	3.78 (2.14–6.66)	2.43 (1.26–4.71)	2.87 (1.67–4.93)	4.12 (2.07–8.23)	<0.0001
AMBP	1.01 ± 0.32	1.23 ± 0.40	2.21 (1.51–3.23)	1.76 (1.01–3.06)	3.23 (1.23–8.51)	1.46 (0.77–2.77)	1.33 (0.65–2.71)	0.43
APOA4	1.03 ± 0.42	1.34 ± 0.59	1.93 (1.42–2.64)	1.36 (0.87–2.11)	1.24 (0.74–2.09)	1.29 (0.77–2.16)	1.08 (0.66–1.77)	0.75
APOB	1.02 ± 0.36	1.15 ± 0.35	1.49 (1.13–1.97)	1.61 (1.17–2.23)	2.05 (1.22–3.45)	1.47 (1.04–2.09)	1.41 (0.85–2.33)	0.19
APOC1	1.01 ± 0.54	1.19 ± 0.53	1.45 (1.06–1.98)	1.59 (1.09–2.31)	2.56 (1.47–4.47)	1.69 (1.12–2.55)	1.18 (0.78–1.77)	0.43
APOC2	1.03 ± 0.62	1.26 ± 0.67	1.60 (1.19–2.14)	1.59 (1.16–2.20)	5.27 (2.30–12.05)	1.48 (1.05–2.08)	1.98 (0.92–4.27)	0.080
APOC3	1.04 ± 0.62	1.33 ± 0.66	1.83 (1.40–2.40)	1.59 (1.16–2.19)	3.55 (1.48–8.50)	1.50 (1.10–2.21)	0.75 (0.46–1.23)	0.26
B2M	1.01 ± 1.31	1.52 ± 1.49	1.75 (1.27–2.41)	1.07 (0.58–2.01)	1.40 (0.65–2.99)	1.01 (0.52–1.95)	0.73 (0.36–1.48)	0.39
ITIH4	1.01 ± 0.26	1.12 ± 0.26	1.57 (1.18–2.09)	1.72 (1.18–2.52)	2.38 (1.31–4.35)	1.46 (0.89–2.39)	1.32 (0.86–2.04)	0.20
PLTP	1.01 ± 0.35	1.13 ± 0.39	1.43 (1.03–2.00)	1.10 (0.73–1.66)	1.13 (0.65–1.98)	1.06 (0.69–1.62)	0.90 (0.60–1.34)	0.60
PTGDS	1.01 ± 1.49	1.57 ± 1.86	1.66 (1.20–2.30)	0.85 (0.44–1.64)	1.18 (0.52–2.65)	1.19 (0.62–2.31)	0.64 (0.32–1.25)	0.64
RBP4	1.01 ± 0.46	1.36 ± 0.65	1.98 (1.40–2.80)	1.71 (1.11–2.64)	3.92 (2.06–7.45)	1.57 (1.001–2.47)	1.28 (0.80–2.04)	0.31

The total number of subjects was 181. There were 145 subjects in the T1DM subcohort group, and a total of 47 T1DM subjects had a CVD event, defined as a first nonfatal myocardial infarction, coronary revascularization, or death from coronary artery disease in CACTI (including 11 CVD subjects in the cohort group). The level of each serum protein from 134 cohort subjects without CVD events was defined as an arbitrary unit of 1. Levels are means ± SDs. Hazard ratios, 95% CI, and *P* values of serum proteins are calculated by Cox proportional hazards models (47 subjects with events and 181 total subjects) using a case-cohort design and the “PROC PHREG” function in SAS with weighting (see Materials and Methods section for details). To improve the model fitting, hazard ratios for CVD events are per SD increase in log-transformed levels of serum proteins. Proteins significantly associated with incident CVD after controlling the Benjamini-Hochberg false discovery rate at 5% are shown. See supplemental Table S1 for protein names. Model 1 is a model adjusted for age, sex, DM duration, and eGFR (the base model). Note: For AMBP, APOA4, B2M, and PTGDS, an interaction term with eGFR was included in each of the models. Model 2 is model 1 further adjusted for nonlipid clinical confounders, including current smoking status, SBP, DBP, levels of HbA1c, log-transformed ACR. For AMBP, APOA4, B2M, and PTGDS, an interaction term with eGFR was included in each of the models. For APOC2 and APOC3, an interaction term with HbA1c was included in each of the models. Model 3 is model 1 further adjusted for medications: statin use and antihypertensive medications. For AMBP, APOA4, and B2M, an interaction term with eGFR was included in each of the models; for PTGDS, an interaction term with on statin medication was included in the model. Model 4 is model 1 further adjusted for lipid risk factors, including HDL-C, LDL-C, and log-transformed TGs. For AMBP, an interaction term with eGFR was included in the model; for APOB and APOC2, an interaction term with log-transformed TG levels was included in each of the models. A2M and APOC3 were adjusted for each other in all adjusted models 1–4, and an interaction term between A2M and APOC3 was also included in model 1.

A2M, alpha-2-macroglobulin; CVD, cardiovasular disease; T1DM, type 1 diabetes mellitus.

A2M and APOC3 were adjusted for each other in all adjusted models 1–4, and an interaction term between A2M and APOC3 was also included in model 1. A2M was significantly associated with incident CVD when adjusting for APOC3 levels in all four models (Table 4). The association between APOC3 and incident CVD was also significant when adjusting for A2M levels in models 1 to 3. However, the APOC3 was no longer associated with incident CVD in model 4 when log-transformed TG was also included as a confounder (Table 4).

**Fig. 2:**
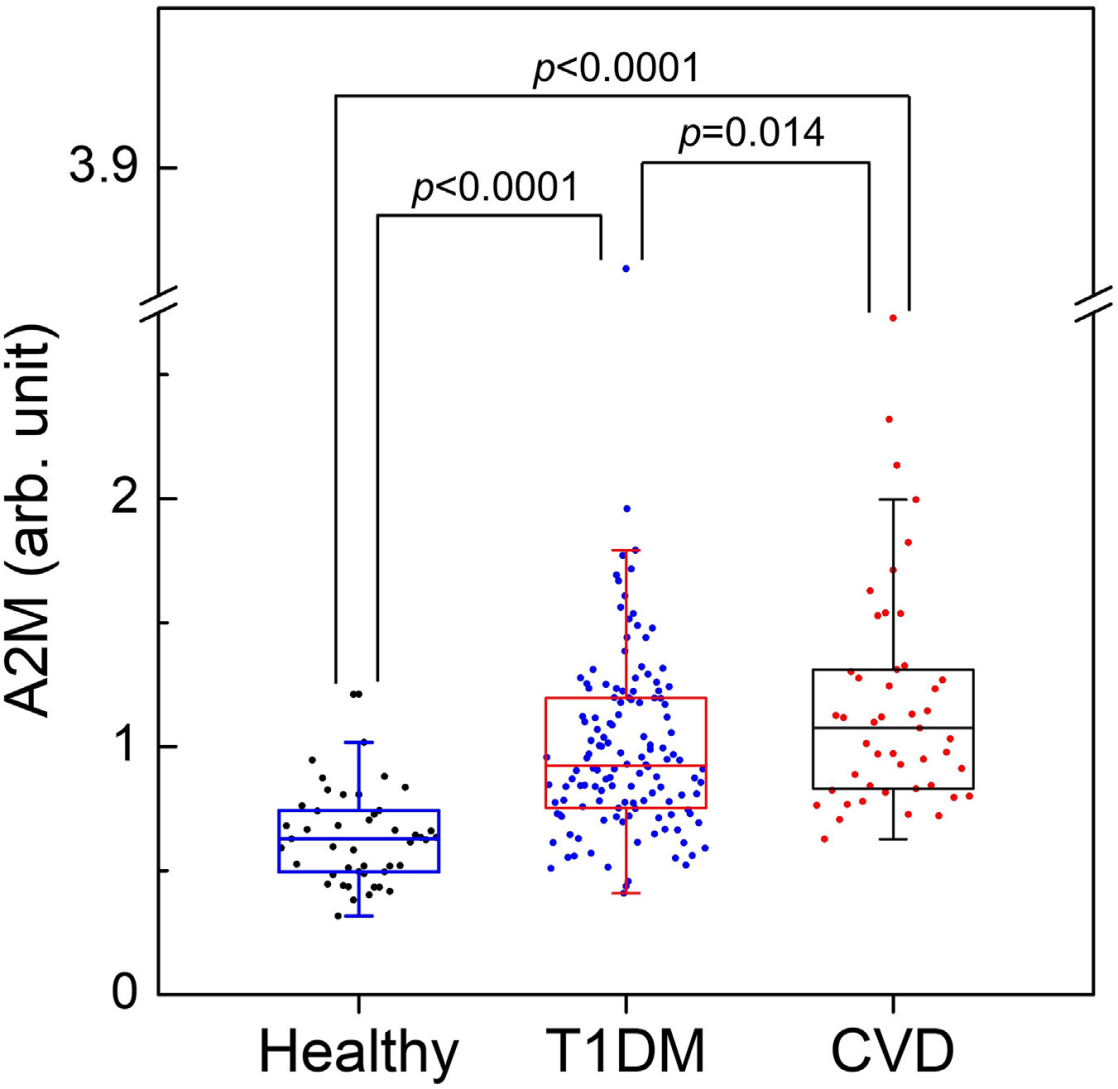
Serum A2M levels in healthy control subjects and T1DM subjects with or without incident CVD in CACTI. Tryptic digests of serum were analyzed using isotope dilution-MS/MS with PRM to quantify A2M levels in three groups: healthy nondiabetic subjects (N = 47), T1DM subjects without CVD events (N = 134), and T1DM subjects with incident CVD (N = 47). The average A2M level in the 134 T1DM subjects without CVD was set to 1.00 in arbitrary units. Box plots show the distribution of A2M levels (median and interquartile ranges), with dots representing individual data points. *P* values from Tukey’s honest sigficant difference post hoc test, following a one-way ANOVA, are provided for comparisons between groups. A2M, alpha-2-macroglobulin; T1DM, type 1 diabetes mellitus; CACTI, Coronary Artery Calcification in Type 1 Diabetes; PRM, parallel reaction monitoring.

In summary, our observations indicate that A2M levels were elevated in T1DM patients compared with healthy controls and were even higher in T1DM patients who later experienced CVD events (Fig. 2).

## Discussion

We first used isotope dilution PRM to quantify 28 serum proteins in subjects with and without T1DM in the CACTI study. In three of the four linear regression models that were adjusted for potential confounders, we found that levels of 10 proteins differed between the two groups. However, in the model adjusted for LDL-C, HDL-C, and log-transformed TGs, only five proteins (A2M, APOA4, APOL1, IGF2, and PLTP) remained significantly different between nondiabetic control subjects and T1DM subjects. Three proteins (A2M, APOA4, and PLTP) had positive associations, whereas two proteins (APOL1 and IGF2) had negative associations. Of the five proteins that significantly associated with T1DM status in all four of our models, A2M exhibited the largest increase (>50%) in relative concentration.

We found that two proteins, APOL1 and IGF2, were negatively associated with T1DM status, whereas three proteins, A2M, APOA4, and PLTP, were positively associated with T1DM status. Notably, three of these proteins—APOA4, APOL1, and PLTP—are lipid related. Previous studies demonstrated that levels of APOA4 were higher in young people with T1DM, and the levels were closely related to glycemic control (33) and positively associated with HbA1c (34). In contrast to the negative association between serum APOL1 levels and T1DM status in the current study, the plasma level of APOL1 was positively associated with a higher risk for incident T2DM after adjustment for confounding variables (35). Consistent with our study, previous research has shown that PLTP, which is involved in the transport of phospholipids, cholesterol, and tocopherols, has elevated activity in patients with T1DM (36, 37, 38, 39).

We next determined whether levels of the 28 serum proteins associated with incident CVD in T1DM subjects in a randomly selected cohort of CACTI. Unadjusted Cox proportional hazards analyses found that 12 proteins associated with incident CVD. However, when we constructed four models that adjusted for potential confounders, only A2M consistently showed a significant positive association with incident CVD. We previously found that APOC3 levels correlated with incident CVD and coronary artery calcium in T1DM (15, 16). In the current analysis, the association with APOC3 was lost after adjusting for TGs and A2M. Thus, A2M exhibited the greatest relative increase in serum levels in T1DM subjects compared with nondiabetic control subjects, and it was the only protein that associated with incident CVD in all our models.

Of the five proteins that remained significantly associated with T1DM status, A2M exhibited the largest increase (>50%) in relative concentration, and it is the only protein in the current study linking T1DM status with CVD risk. Previous studies on cohorts without T1DM show that high levels of plasma A2M associate with CVD risk (40), and HDL subspecies containing A2M associated with higher coronary heart disease risk in four prospective studies (41).

A2M is an abundant glycoprotein known for its role as a broad-spectrum protease inhibitor (42, 43). It can inhibit and neutralize various classes of proteases, including aspartic, serine-, cysteine-, and metalloproteinases. The protein helps regulate several physiological processes, particularly those related to inflammation and immunity (43). Alterations in serum levels of A2M occur across a spectrum of inflammatory and immune diseases (43). However, interpreting these findings is challenging because of discrepancies in assay methodologies among different studies and inconsistent measurements of protein levels, which have been reported to vary over six orders of magnitude (44). It is essential to consider these limitations when interpreting the results of studies investigating A2M levels in various disease states.

Consistent with our findings, previous studies demonstrated that A2M levels increase in patients with T1DM (45). Higher levels of A2M were also found in another study of individuals with diabetes compared with nondiabetic controls (44). However, most of the cases in that study had T2DM, and the analysis did not adjust for diabetes type or other potential clinical confounders, which could influence the interpretation of the results. A large study using a validated method highlighted that A2M circulates predominantly as a tetramer. In contrast, our study quantified A2M levels with a highly specific MS/MS method that can detect all forms of A2M present in serum. Importantly, our analyses were adjusted for a wide range of potential confounders. These methodological details underscore the rigor of our approach and enhance confidence in the reported findings.

It is important to note that diabetic kidney disease associates with increased CVD risk and that A2M associates with diabetic kidney disease (46). Therefore, in our analyses of the links between serum proteins and incident CVD, we included eGFR in the base model and further included ACR in model 2. Moreover, in the analysis of the association between serum proteins and T1DM status, we included both eGFR and ACR in model 2. We found that the association of A2M with T1DM status and CVD risk was independent of eGFR and ACR.

The role of A2M in various cardiovascular and thrombotic conditions is complex. Increased levels of A2M are associated with thrombosis, atherosclerosis development, and cardiac hypertrophy, although the underlying mechanisms are not fully understood. In nephrotic syndrome, A2M was reported to exhibit both anticoagulant and procoagulant activity, reflecting its intricate involvement in hemostasis and coagulation pathways (47, 48). In children with symptomatic thromboembolism, elevated A2M levels were independently linked to increased odds of stroke and deep-vein thrombosis (49), indicating the potential role of A2M as a marker for thrombotic events in that population.

Inflammation may be a key factor linking higher serum levels of A2M to the risk of CVD. Interleukin-6, a proinflammatory cytokine implicated in cerebrovascular disease and CVD binds A2M (26). This suggests that A2M might contribute to the development of CVD by participating in various inflammatory pathways through its interaction with interleukin-6. Furthermore, it is possible that A2M may play a role in the progression of atherosclerotic lesions by interacting with low-density lipoprotein receptor-related protein 1 (27) as A2M competes with lipoprotein lipase for binding to low-density lipoprotein receptor-related protein 1 (50). The increased levels of A2M in T1DM may prevent atherogenic remnant lipoproteins from being cleared properly. This mechanism is less likely because A2M did not associate with plasma lipid levels in our study.

Strengths of our study include a well-characterized subcohort of T1DM subjects and subjects without diabetes who were enrolled in CACTI, a validated MS/MS approach for quantifying serum proteins, and stringent statistical analyses. Our study also has limitations, however. First, there were a modest number of T1DM subjects with incident CVD despite the large number of subjects enrolled in CACTI. Second, our studies are associational and do not demonstrate causality. It will be important to confirm and extend our findings in larger cohorts of T1DM patients and to extend our observations to patients with T2DM. It will also be critical to investigate the molecular mechanisms underlying the association of increased levels of serum A2M with diabetes and incident CVD risk in T1DM patients and to determine whether A2M plays a causal role in atherosclerosis.

In conclusion, we found that A2M levels are elevated in subjects with T1DM compared with nondiabetic subjects. Moreover, of the 28 serum proteins we quantified, only A2M consistently and independently associated with incident CVD risk in T1DM subjects. These findings suggest that A2M could serve as both a serum marker and potentially a mediator of CVD risk in people with T1DM.

## Data availability

The data supporting this study are available in the article, the supplemental Data, or from the corresponding author upon reasonable request. The raw MS data have been deposited at the Panorama server (https://panoramaweb.org/CACTISerum.url).

## Supplemental data

This article contains supplemental data (15, 21, 22, 23, 24, 29, 30, 31, 32).

## Conflict of interest

The authors declare that they have no conflicts of interest with the contents of this article.

## References

[1] GBD 2021 Diabetes Collaborators (2023). Global, regional, and national burden of diabetes from 1990 to 2021, with projections of prevalence to 2050: a systematic analysis for the Global Burden of Disease Study 2021. Lancet.

[2] Semenkovich C.F. (2017). We know more than we can tell about diabetes and vascular disease: the 2016 edwin bierman award lecture. Diabetes.

[3] Peterson L.R., McKenzie C.R., Schaffer J.E. (2012). Diabetic cardiovascular disease: getting to the heart of the matter. J. Cardiovasc. Transl. Res..

[4] Orchard T.J., Nathan D.M., Zinman B., Cleary P., Brillon D., Backlund J.Y. (2015). Association between 7 years of intensive treatment of type 1 diabetes and long-term mortality. JAMA.

[5] Schofield J., Ho J., Soran H. (2019). Cardiovascular risk in type 1 diabetes mellitus. Diabetes Ther..

[6] Marcovecchio M.L. (2020). Importance of identifying novel biomarkers of microvascular damage in type 1 diabetes. Mol. Diagn. Ther..

[7] Kontush A. (2016). Identifying new risk markers and potential targets: the value of the proteome. Cardiovasc. Drugs Ther..

[8] Lam M.P., Ping P., Murphy E. (2016). Proteomics research in cardiovascular medicine and biomarker discovery. J. Am. Coll. Cardiol..

[9] Anderson N.L., Anderson N.G. (2002). The human plasma proteome: history, character, and diagnostic prospects. Mol. Cell Proteomics.

[10] Shao B., Heinecke J.W. (2018). Quantifying HDL proteins by mass spectrometry: how many proteins are there and what are their functions?. Expert Rev. Proteomics.

[11] Yin X., Baig F., Haudebourg E., Blankley R.T., Gandhi T., Muller S. (2017). Plasma proteomics for epidemiology: increasing throughput with standard-flow rates. Circ. Cardiovasc. Genet..

[12] Piarulli F., Banfi C., Ragazzi E., Gianazza E., Munno M., Carollo M. (2024). Multiplexed MRM-based proteomics for identification of circulating proteins as biomarkers of cardiovascular damage progression associated with diabetes mellitus. Cardiovasc. Diabetol..

[13] Bourmaud A., Gallien S., Domon B. (2016). Parallel reaction monitoring using quadrupole-Orbitrap mass spectrometer: principle and applications. Proteomics.

[14] Ronsein G.E., Pamir N., von Haller P.D., Kim D.S., Oda M.N., Jarvik G.P. (2015). Parallel reaction monitoring (PRM) and selected reaction monitoring (SRM) exhibit comparable linearity, dynamic range and precision for targeted quantitative HDL proteomics. J. Proteomics.

[15] Kanter J.E., Shao B., Kramer F., Barnhart S., Shimizu-Albergine M., Vaisar T. (2019). Increased apolipoprotein C3 drives cardiovascular risk in type 1 diabetes. J. Clin. Invest..

[16] Buckner T., Shao B., Eckel R.H., Heinecke J.W., Bornfeldt K.E., Snell-Bergeon J. (2021). Association of apolipoprotein C3 with insulin resistance and coronary artery calcium in patients with type 1 diabetes. J. Clin. Lipidol..

[17] Mehta A., Shapiro M.D. (2022). Apolipoproteins in vascular biology and atherosclerotic disease. Nat. Rev. Cardiol..

[18] Green P.S., Vaisar T., Pennathur S., Kulstad J.J., Moore A.B., Marcovina S. (2008). Combined statin and niacin therapy remodels the high-density lipoprotein proteome. Circulation.

[19] He Y., Ronsein G.E., Tang C., Jarvik G.P., Davidson W.S., Kothari V. (2020). Diabetes impairs cellular cholesterol efflux from ABCA1 to small HDL particles. Circ. Res..

[20] Ronsein G.E., Reyes-Soffer G., He Y., Oda M., Ginsberg H., Heinecke J.W. (2016). Targeted proteomics identifies paraoxonase/arylesterase 1 (PON1) and apolipoprotein Cs as potential risk factors for hypoalphalipoproteinemia in diabetic subjects treated with fenofibrate and rosiglitazone. Mol. Cell Proteomics.

[21] Shao B., de Boer I., Tang C., Mayer P.S., Zelnick L., Afkarian M. (2015). A cluster of proteins implicated in kidney disease is increased in high-density lipoprotein isolated from hemodialysis subjects. J. Proteome Res..

[22] Shao B., Mathew A.V., Thornock C., Pennathur S. (2021). Altered HDL proteome predicts incident CVD in chronic kidney disease patients. J. lipid Res..

[23] Shao B., Snell-Bergeon J.K., Pyle L.L., Thomas K.E., de Boer I.H., Kothari V. (2022). Pulmonary surfactant protein B carried by HDL predicts incident CVD in patients with type 1 diabetes. J. Lipid Res..

[24] Shao B., Zelnick L.R., Wimberger J., Himmelfarb J., Brunzell J., Davidson W.S. (2019). Albuminuria, the high-density lipoprotein proteome, and coronary artery calcification in type 1 diabetes mellitus. Arterioscler. Thromb. Vasc. Biol..

[25] Vaisar T., Kanter J.E., Wimberger J., Irwin A.D., Gauthier J., Wolfson E. (2020). High concentration of medium-sized HDL particles and enrichment in HDL paraoxonase 1 associate with protection from vascular complications in people with long-standing type 1 diabetes. Diabetes Care.

[26] Vaisar T., Pennathur S., Green P.S., Gharib S.A., Hoofnagle A.N., Cheung M.C. (2007). Shotgun proteomics implicates protease inhibition and complement activation in the antiinflammatory properties of HDL. J. Clin. Invest..

[27] Kothari V., Ho T.W.W., Cabodevilla A.G., He Y., Kramer F., Shimizu-Albergine M. (2024). Imbalance of APOB lipoproteins and large HDL in type 1 diabetes drives atherosclerosis. Circ. Res..

[28] Lima D.B., Melchior J.T., Morris J., Barbosa V.C., Chamot-Rooke J., Fioramonte M. (2018). Characterization of homodimer interfaces with cross-linking mass spectrometry and isotopically labeled proteins. Nat. Protoc..

[29] MacLean B., Tomazela D.M., Shulman N., Chambers M., Finney G.L., Frewen B. (2010). Skyline: an open source document editor for creating and analyzing targeted proteomics experiments. Bioinformatics.

[30] Barlow W.E. (1994). Robust variance estimation for the case-cohort design. Biometrics.

[31] Barbieri S., Mehta S., Wu B., Bharat C., Poppe K., Jorm L. (2022). Predicting cardiovascular risk from national administrative databases using a combined survival analysis and deep learning approach. Int. J. Epidemiol..

[32] Kazemi A., Sasani N., Mokhtari Z., Keshtkar A., Babajafari S., Poustchi H. (2022). Comparing the risk of cardiovascular diseases and all-cause mortality in four lifestyles with a combination of high/low physical activity and healthy/unhealthy diet: a prospective cohort study. Int. J. Behav. Nutr. Phys. Act..

[33] Attia N., Touzani A., Lahrichi M., Balafrej A., Kabbaj O., Girard-Globa A. (1997). Response of apolipoprotein AIV and lipoproteins to glycaemic control in young people with insulin-dependent diabetes mellitus. Diabet Med..

[34] Quilliot D., Walters E., Guerci B., Fruchart J.C., Duriez P., Drouin P. (2001). Effect of the inflammation, chronic hyperglycemia, or malabsorption on the apolipoprotein A-IV concentration in type 1 diabetes mellitus and in diabetes secondary to chronic pancreatitis. Metabolism.

[35] Croyal M., Wargny M., Chemello K., Chevalier C., Blanchard V., Bigot-Corbel E. (2022). Plasma apolipoprotein concentrations and incident diabetes in subjects with prediabetes. Cardiovasc. Diabetol..

[36] de Vries R., Kerstens M.N., Sluiter W.J., Groen A.K., van Tol A., Dullaart R.P. (2005). Cellular cholesterol efflux to plasma from moderately hypercholesterolaemic type 1 diabetic patients is enhanced, and is unaffected by simvastatin treatment. Diabetologia.

[37] Lassenius M.I., Makinen V.P., Fogarty C.L., Peraneva L., Jauhiainen M., Pussinen P.J. (2014). Patients with type 1 diabetes show signs of vascular dysfunction in response to multiple high-fat meals. Nutr. Metab. (Lond.).

[38] Colhoun H.M., Taskinen M.R., Otvos J.D., Van Den Berg P., O'Connor J., Van Tol A. (2002). Relationship of phospholipid transfer protein activity to HDL and apolipoprotein B-containing lipoproteins in subjects with and without type 1 diabetes. Diabetes.

[39] Colhoun H.M., Scheek L.M., Rubens M.B., Van Gent T., Underwood S.R., Fuller J.H. (2001). Lipid transfer protein activities in type 1 diabetic patients without renal failure and nondiabetic control subjects and their association with coronary artery calcification. Diabetes.

[40] de Laat-Kremers R., Costanzo S., Yan Q., Di Castelnuovo A., De Curtis A., Cerletti C. (2024). High alpha-2-macroglobulin levels are a risk factor for cardiovascular disease events: a Moli-sani cohort study. Thromb. Res..

[41] Sacks F.M., Liang L., Furtado J.D., Cai T., Davidson W.S., He Z. (2020). Protein-defined subspecies of HDLs (High-Density lipoproteins) and differential risk of coronary heart disease in 4 prospective studies. Arterioscler Thromb. Vasc. Biol..

[42] Sottrup-Jensen L. (1989). Alpha-macroglobulins: structure, shape, and mechanism of proteinase complex formation. J. Biol. Chem..

[43] Vandooren J., Itoh Y. (2021). Alpha-2-Macroglobulin in inflammation, immunity and infections. Front. Immunol..

[44] Yoshino S., Fujimoto K., Takada T., Kawamura S., Ogawa J., Kamata Y. (2019). Molecular form and concentration of serum alpha(2)-macroglobulin in diabetes. Sci. Rep..

[45] do Nascimento de Oliveira V., Lima-Neto A.B.M., van Tilburg M.F., de Oliveira Monteiro-Moreira A.C., Duarte Pinto Lobo M., Rondina D. (2018). Proteomic analysis to identify candidate biomarkers associated with type 1 diabetes. Diabetes Metab. Syndr. Obes..

[46] Trink J., Li R., Palarasah Y., Troyanov S., Andersen T.E., Sidelmann J.J. (2021). Activated alpha 2-macroglobulin is a novel mediator of mesangial cell profibrotic signaling in diabetic kidney disease. Biomedicines.

[47] Hoogendoorn H., Toh C.H., Nesheim M.E., Giles A.R. (1991). Alpha 2-macroglobulin binds and inhibits activated protein C. Blood.

[48] Mitchell L., Piovella F., Ofosu F., Andrew M. (1991). Alpha-2-macroglobulin may provide protection from thromboembolic events in antithrombin III-deficient children. Blood.

[49] Beheiri A., Langer C., During C., Krumpel A., Thedieck S., Nowak-Gottl U. (2007). Role of elevated alpha2-macroglobulin revisited: results of a case-control study in children with symptomatic thromboembolism. J. Thromb. Haemost..

[50] Chappell D.A., Fry G.L., Waknitz M.A., Iverius P.H., Williams S.E., Strickland D.K. (1992). The low density lipoprotein receptor-related protein/alpha 2-macroglobulin receptor binds and mediates catabolism of bovine milk lipoprotein lipase. J. Biol. Chem..

