# Supplementary material for: Elevated levels of serum alpha-2-macroglobulin associate with diabetes status and incident CVD in type 1 diabetes
Source: J Lipid Res. 2025 Jan 4;66(2):100741. doi: 10.1016/j.jlr.2025.100741 (PMC11841089; doi:10.1016/j.jlr.2025.100741)
Supplement: Supplemental Tables S1–S3 [file mmc1.pdf]

Elevated Levels of Serum Alpha-2-Macroglobulin Associate with Diabetes Status and Incident Cardiovascular Disease in Type 1 Diabetes

Baohai Shao[1], Janet K. Snell-Bergeon[2], Ian H. de Boer[1], W. Sean Davidson[3], Karin E. Bornfeldt[1], and Jay W. Heinecke[1]

[1]Department of Medicine, University of Washington, Seattle, WA 98109, [2]Barbara Davis Center for Diabetes, University of Colorado Anschutz Medical Campus, Aurora, CO, 80045, [3]Department of Pathology and Laboratory Medicine, University of Cincinnati, Cincinnati, OH 45237

SUPPLEMENTAL METHODS

Serum protein digestion. In a dilution plate, 5 µL serum was added into 245 µL of 25 mM NH_4HCO_3. In a digestion plate, 10 µL of the diluted serum (containing 0.2 µL of serum and ~14 µg of serum proteins) was placed in each well. Following the addition of freshly prepared methionine (Met, 5 mM final concentration) in 20% acetonitrile and 100 mM NH_4HCO_3, serum proteins (~14 µg) were reduced with dithiothreitol (2.5 mM final concentration, 45 min at 60°C) and then alkylated with iodoacetamide (5 mM final concentration, 30 min at room temperature). After adding 0.2 µg of isotope-labeled [^{15}N]APOA1 (as the internal standard), we incubated the serum overnight (20 h) at 37°C with 25:1 (w/w, proteins/enzyme) sequencing-grade modified trypsin (Promega). Digestion was halted by acidifying the reaction mixture (pH 2 to 3) with 5 µL of 20% trifluoroacetic acid. Then the samples were dried and stored at −80°C until LC-MS analysis.

Liquid chromatography-electrospray ionization tandem mass spectrometric (LC-ESI-MS/MS) analysis of serum proteins by parallel reaction monitoring. To quantitatively measure relative levels of the serum proteins, we used targeted proteomics with parallel reaction monitoring (PRM) as previously reported (1, 2). Briefly, a nanoACQUITY UPLC (Waters, Milford, MA) was used for the separation, with a multistep gradient of 0.1% formic acid in water (solvent A) and 0.1% formic acid in acetonitrile (solvent B). After the dried peptide digests were reconstituted in 0.1% formic acid and 1% acetonitrile in water, serum peptide digests (equivalent to 0.2 µg of protein) were desalted on a C18 trap column (0.1 × 40 mm, packed in house with Magic C-18 reverse-phase resin, 5 µm; 100 Å; Michrom Bioresources) for 8 min at 2.5 µL/min in 99% solvent A. They were then separated using a C-18 analytical column (0.1 × 150 mm, packed in house with Magic C-18 reverse-phase resin, 5 µm; 100 Å; Michrom Bioresources) with an uncoated SilicaTip Emitters (20 µm ID, 10 µm tip ID; New Objective, Woburn, MA). The column was kept at 50°C, and the peptides were eluted from the trap column onto the analytical column at a flow rate of 0.5 µL/min. They were separated using the following multistep gradient: 1% to 10% solvent B in 2 min; 10% to 25% solvent B in 20 min; 25% to 35% solvent B in 5 min; and 35% to 80% solvent B in 3 min. The column was subsequently washed for 2 min in 80% solvent B; from 80% to 1% solvent B in 1 min; and re-equilibrated in 1% solvent B for 13 min. The peptide digests were then analyzed with an ultrahigh-resolution accurate mass Orbitrap Fusion

Lumos Tribrid Mass Spectrometer (Thermo Fisher Scientific, San Jose, CA) operated in the PRM mode, using 2100 V of spray voltage and 300°C ion transfer tube temperature. The acquisition method combined a full scan (MS1) method with a time scheduled sequential PRM (MS2) method. For the full MS1 scans, a scan range of m/z 350-1800, an Orbitrap resolution of 120,000, a target automatic gain control (AGC) value of 200,000, and a maximum injection time of 50 ms were used. For the PRM MS2 scans, using the list of target proteins described in the "Protein selection" section, we isolated and fragmented precursor ions with charge state 2 or 3 by using higher energy collisional dissociation (HCD) fragmentation with 30% collision energy with a stepped collision energy of 5%. The retention time (RT) of each targeted peptide was determined in preliminary PRM test runs, and the scheduled time window was targeted in RT±1.6 minutes. A scan range of m/z 120-2,000 and mass resolution of 15,000 with a maximum injection time of 20 ms and target AGC of 10,000 were used.

The peptides for each protein were selected from those detected by shotgun analysis and from our previous studies (3, 4). For quantification, two or more peptides were selected for 24 proteins, and one peptide for four proteins (**Supplemental Table 1**). The selected peptides were reliably detected, and the relative abundances of peptides from the same protein correlated highly when protein concentration was varied. All peptides selected for each protein were unique to that protein with the exception of SAA1, where we used one peptide shared by SAA1 and SAA2, and SAA2 was not included in the current study because we detected only very low levels of other SAA2 peptides. Because oxidation of methionine residues might affect quantification, we avoided methionine-containing peptides.

Quantifying serum proteins with ^{15}N-labeled APOA1 as the internal standard. The targeted PRM MS data of peptides from each serum protein were analyzed using Skyline (version 23.1.0.380), an open-source program (5). Before digestion, we added an equal amount of ^{15}N-labeled APOA1 to each serum sample as an internal standard. The peak areas of all the transitions of a peptide detected by PRM were summed to get the total peak area for the peptide. Transitions with interferences were omitted from the analyses. To normalize the peak area of a peptide, the total peak area of all selected transitions of the peptide was divided by the peak area of each of the three ^{15}N-labeled peptides from ^{15}N-APOA1 (**Supplemental Table 1**), and the ratios were used for quantification (6). To calculate relative levels of a peptide, we first calculated the average ratio of that peptide to one of the three ^{15}N-APOA1 peptides in T1DM subjects without CVD, then the level of each subject was divided by that average ratio, with the new ratio representing the relative level of each subject. Next**,** the three new ratios (from comparison to three ^{15}N-labeled peptides from ^{15}N-APOA1) were averaged. If two or more peptides were quantified for a protein, their relative levels were averaged to obtain the relative level of that protein in serum.

Statistical analysis. The values of clinical characteristics are median and interquartile ranges (IQR) for continuous covariates and N (%) for categorical covariates. *P*-values are from Student's *t*-test (for normally distributed variables), the Mann-Whitney U test (for non-normally distributed variables), or the Pearson Chi-Square test (for categorical

variables). Difference of serum protein levels between healthy controls and T1DM patients, 95% confidence intervals (CI), and P-values are calculated by linear regression, in which the protein level was used as dependent variable and T1DM status was used as an independent variable.

For the association between serum protein levels and incident CVD, hazard ratios, 95% confidence intervals (CI), and p-values were calculated using Cox proportional hazards models in a case-cohort design. The analysis included all 47 CVD events and 181 total subjects, with a subcohort of 145 subjects (26.5% of the full cohort). The risk set at each event time included the case and members of the subcohort who were at risk. Cases outside the subcohort were only in the risk set for their event time. Using SAS “PROC PHREG”, regression coefficients and robust variance estimates were computed with Breslow’s method (7). Controls in the subcohort were weighted inversely by the sampling fraction (1/0.265 = 3.77), while cases had a weight of 1 at the time of the event. Hazard ratios were calculated for CVD events per SD increase in log-transformed serum protein levels to improve model fitting.

To account for multiple comparison testing of serum proteins, we first used the Benjamini-Hochberg method with a 5% false discovery rate. Based on corrected or adjusted *p*-values (*q*-values), only proteins with a *q*-value <0.05 were initially considered significant. Then models were constructed to adjust for potential confounding variables. Because there were only 47 CVD cases and 47 healthy control subjects, we built sequential models by including each group of confounders in a model one at a time to avoid overfitting the data.

If the level of a protein interacted significantly with a clinical parameter, the interaction term was included in the adjusted model (8, 9). The main reason for including interaction terms is to test for “effect modification”, where the effect of one variable on the outcome (time to events) may depend on the level of another variable. When a significant interaction is discovered, it can provide important clinical insights into understanding how different factors work together to influence time to events. By adding interaction terms, the model can better capture the complex relationships between variables, thereby representing the data more accurately, even though interpreting interaction terms can be more complicated than main effects.

Two-sided *p*-values <0.05 were considered significant, except for multiple comparison tests, in which case, an adjusted *p*-values (or *q*-values) <0.05 were considered significant using a 5% false discovery rate. All statistical analyses were performed with SPSS (Windows version 19, Chicago, IL) or SAS OnDemand for Academics.

REFERENCES

1. Shao, B., J. K. Snell-Bergeon, L. L. Pyle, K. E. Thomas, I. H. de Boer, V. Kothari, J. Segrest, W. S. Davidson, K. E. Bornfeldt, and J. W. Heinecke. 2022. Pulmonary surfactant protein B carried by HDL predicts incident CVD in patients with type 1 diabetes. *J Lipid Res* **63**: 100196.
2. Kanter, J. E., B. Shao, F. Kramer, S. Barnhart, M. Shimizu-Albergine, T. Vaisar, M. J. Graham, R. M. Crooke, C. R. Manuel, R. A. Haeusler, D. Mar, K. Bomsztyk, J. E. Hokanson, G. L. Kinney, J. K. Snell-Bergeon, J. W. Heinecke, and K. E. Bornfeldt. 2019. Increased apolipoprotein C3 drives cardiovascular risk in type 1 diabetes. *J Clin Invest* **130**: 4165-4179.
3. Shao, B., I. de Boer, C. Tang, P. S. Mayer, L. Zelnick, M. Afkarian, J. W. Heinecke, and J. Himmelfarb. 2015. A Cluster of Proteins Implicated in Kidney Disease Is Increased in High-Density Lipoprotein Isolated from Hemodialysis Subjects. *J Proteome Res* **14**: 2792-2806.
4. Shao, B., L. R. Zelnick, J. Wimberger, J. Himmelfarb, J. Brunzell, W. S. Davidson, J. K. Snell-Bergeon, K. E. Bornfeldt, I. H. de Boer, and J. W. Heinecke. 2019. Albuminuria, the High-Density Lipoprotein Proteome, and Coronary Artery Calcification in Type 1 Diabetes Mellitus. *Arteriosclerosis, thrombosis, and vascular biology* **39**: 1483-1491.
5. MacLean, B., D. M. Tomazela, N. Shulman, M. Chambers, G. L. Finney, B. Frewen, R. Kern, D. L. Tabb, D. C. Liebler, and M. J. MacCoss. 2010. Skyline: an open source document editor for creating and analyzing targeted proteomics experiments. *Bioinformatics* **26**: 966-968.
6. Shao, B., A. V. Mathew, C. Thornock, and S. Pennathur. 2021. Altered HDL Proteome Predicts Incident CVD in Chronic Kidney Disease Patients. *Journal of lipid research*: 100135.
7. Barlow, W. E. 1994. Robust variance estimation for the case-cohort design. *Biometrics* **50**: 1064-1072.
8. Barbieri, S., S. Mehta, B. Wu, C. Bharat, K. Poppe, L. Jorm, and R. Jackson. 2022. Predicting cardiovascular risk from national administrative databases using a combined survival analysis and deep learning approach. *Int J Epidemiol* **51**: 931-944.
9. Kazemi, A., N. Sasani, Z. Mokhtari, A. Keshtkar, S. Babajafari, H. Poustchi, M. Hashemian, and R. Malekzadeh. 2022. Comparing the risk of cardiovascular diseases and all-cause mortality in four lifestyles with a combination of high/low physical activity and healthy/unhealthy diet: a prospective cohort study. *Int J Behav Nutr Phys Act* **19**: 138.

Supplemental Table 1. Peptides Monitored for Each Protein by PRM Analysis.

Proteins	Description	Peptides	Precursor (m/z)	Charge State	RT (min)
A2M	Alpha-2-macroglobulin	IAQWQSFQLEGGLK	802.9252	2	23.5
A2M		LLIYAVLPTGDVIGDSAK	923.022	2	30.3
AMBP	Alpha-1-Microglobulin/Bikunin Precursor	GECVPGEQEPEPILIPR	960.4802	2	21.6
AMBP		TVAACNLPIVR	607.3397	2	17.8
AMBP		EYCGVPGDGDEELLR	854.8778	2	18.4
^{15}N-APOA1	^{15}N-labeled Apolipoprotein A-I	DYVSQFEGSALGK	708.3160	2	20.9
^{15}N-APOA1		VQPYLDDFQK	633.2948	2	17.1
^{15}N-APOA1		THLAPYSDELR	659.3041	2	12.5
APOA1	Apolipoprotein A-I	DYVSQFEGSALGK	700.8383	2	20.9
APOA1		VQPYLDDFQK	626.8141	2	17.1
APOA1		THLAPYSDELR	651.3279	2	12.5
APOA1		AKPALEDLR	506.7929	2	11.7
APOA1		VSFLSALEEYTK	693.8612	2	29.6
APOA2	Apolipoprotein A-II	EPCVESLVSQYFQTVTDYGK	784.0351	3	32.3
APOA2		SPELQAEAK	486.7535	2	10.9
APOA4	Apolipoprotein A-IV	LAPLAEDVR	492.2796	2	15.2
APOA4		LEPYADQLR	552.7878	2	14.3
APOA4		LGEVNTYAGDLQK	704.3594	2	14.9
APOA4		IDQNVEELK	544.2851	2	12.1
APOA4		SELTQQLNALFQDK	817.9229	2	27.4
APOB	Apolipoprotein B	IAELSATAQEIIK	693.8956	2	20.9
APOB		VELEVPQLCSFILK	837.9604	2	31.3
APOB		TEVIPPLIENR	640.8641	2	21.7
APOB		VPSYTLILPSLELPVLHVPR	748.4436	3	32.1
APOB		FPEVDVLTK	524.2897	2	21.1
APOB		ATGVLYDYVNK	621.8219	2	17.5
APOB		EVGTVLSQVYSK	748.4436	3	32.1

Proteins	Description	Peptides	Precursor (m/z)	Charge State	RT (min)
APOC1	Apolipoprotein C-I	EFGNTLEDK	526.7484	2	12.3
APOC1		EWFSETFQK	601.2798	2	20.4
APOC2	Apolipoprotein C-II	ESLSSYWESAK	643.7986	2	18.3
APOC2		TAAQNLYEK	519.2667	2	11.0
APOC2		TYLPAVDEK	518.2715	2	14.5
APOC3	Apolipoprotein C-III	DALSSVQESQVAQQAR	858.9292	2	14.2
APOC3		DALSSVQESQVAQQAR	572.9552	3	14.2
APOC3		GWVTDGFSSLK	598.8009	2	22.2
APOC4	Apolipoprotein C-IV	ELLETVVNR	536.8035	2	17.5
APOE	Apolipoprotein E	LAVYQAGAR	474.7667	2	12.0
APOE		LGPLVEQGR	484.7798	2	13.9
APOE		SELEEQLTPVAEETR	865.9258	2	17.7
APOE		AATVGSLAGQPLQER	749.4046	2	15.4
APOL1	Apolipoprotein L-I	LNILNNNYK	553.3037	2	16.5
APOL1		VAQELEEK	473.2480	2	10.8
APOM	Apolipoprotein M	WIYHLTEGSTDLR	530.9352	3	18.2
APOM		SLTSCLDSK	505.7448	2	12.3
APOM		AFLLTPR	409.2502	2	19.4
B2M	Beta-2-Microglobulin	SNFLNCYVSGFHPSDIEVDLLK	852.0809	3	29.4
B2M		VEHSDLSFSK	383.5243	3	11.3
CETP	Cholesteryl ester transfer protein	GVSLFDIINPEIITR	843.9749	2	32.9
CETP		LFLSLLDFQITPK	767.9476	2	33.1
HPR	Haptoglobin-related protein	VGYVSGWGQSDNFK	772.3624	2	18.1
IGF2	Insulin-like growth factor II	GIVEECCFR	585.2575	2	14.1
IGF2		SCDLALLETYCATPAK	906.9290	2	22.4
ITIH4	Inter-alpha-trypsin inhibitor heavy chain H4	ILDDLSPR	464.7585	2	15.7
ITIH4		IPKPEASFSPR	410.2277	2	12.2
ITIH4		ITFELVYEELLK	748.9160	2	32.1
LCAT	Lecithin:cholesterol acyltransferase	SSGLVSNAPGVQIR	692.8808	2	16.1

Proteins	Description	Peptides	Precursor (m/z)	Charge State	RT (min)
LCAT		STELCGLWQGR	653.8141	2	19.8
LCAT		LEPGQQEEYYR	706.3281	2	11.8
LCAT		TYSVEYLDSSK	646.3063	2	15.2
LPA	Apolipoprotein(a)	NPDAVAAPYCYTR	749.3432	2	14.4
PLG	Plasminogen	EAQLPVIENK	570.8166	2	15.7
PLG		HSIFTPETNPR	649.8280	2	12.2
PLG		HSIFTPETNPR	433.5544	3	12.2
PLG		LSSPAVITDK	515.7926	2	13.4
PLTP	Phospholipid transfer protein	ATYFGSIVLLSPAVIDSPLK	1046.0904	2	32.6
PLTP		AVEPQLQEEER	664.3281	2	11.7
PON1	Paraoxonase/arylesterase 1	EVQPVELPNCNLVK	819.9296	2	19.8
PON1		IFFYDSENPPASEVLR	942.4623	2	25.0
PON1		SFNPNSPGK	474.2327	2	10.8
PON1		IQNILTEEPK	592.8297	2	16.0
PON3	Paraoxonase/arylesterase 3	STVEIFK	412.2316	2	16.5
PON3		ILIGTVFHK	514.3186	2	19.1
PTGDS	Prostaglandin D2 synthase	AQGFTEDTIVFLPQTDK	955.4807	2	25.7
RBP4	Retinol Binding Protein 4	YWGVASFLQK	599.8164	2	25.8
RBP4		GNDDHWIVDTDYDTYAVQYSCR	898.3785	3	21.7
RBP4		LLNLDGTCADSYSFVFSR	1032.9884	2	28.3
SAA1	Serum Amyloid A1	SFFSFLGEAFDGAR	775.8673	2	32.6
SAA1		GPGGVWAAEAISDAR	728.8626	2	20.3
SAA1		FFGHGAEDSLADQAANEWGR	726.6594	3	19.8
SERPINA1	Alpha-1-antitrypsin	SASLHLPK	426.7505	2	11.7
SERPINA1		LSITGTYDLK	555.8057	2	18.5
SERPINA1		SVLGQLGITK	508.3109	2	20.4
SERPINA1		VFSNGADLSGVTEEAPLK	917.4651	2	20.3
SERPINA1		AVLTIDEK	444.7555	2	13.9

Supplemental Table 2. Unadjusted Associations of Serum Proteins with T1DM Status in CACTI.

Protein	Level (mean±SD) (Arb. Unit)		Unadjusted Difference	95% CI		*P*-Value	Q-Value
	Healthy (N=47)	T1DM (N=134)		Lower	Upper		
**A2M**	**0.64±0.20**	**1±0.40**	**0.36**	**0.24**	**0.48**	**<0.0001**	**<0.0001**
**APOA4**	**0.74±0.25**	**1±0.39**	**0.26**	**0.14**	**0.38**	**<0.0001**	**0.00034**
**APOC2**	**1.38±0.61**	**1±0.56**	**-0.38**	**-0.57**	**-0.19**	**0.00010**	**0.00097**
**PLTP**	**0.80±0.21**	**1±0.34**	**0.20**	**0.09**	**0.30**	**0.00023**	**0.0016**
**APOB**	**1.20±0.32**	**1±0.35**	**-0.20**	**-0.32**	**-0.08**	**0.00079**	**0.0044**
**APOC4**	**1.48±0.96**	**1±0.81**	**-0.48**	**-0.77**	**-0.20**	**0.00097**	**0.0045**
**APOL1**	**1.21±0.51**	**1±0.32**	**-0.21**	**-0.35**	**-0.08**	**0.0018**	**0.0073**
**IGF2**	**1.15±0.31**	**1±0.32**	**-0.15**	**-0.25**	**-0.05**	**0.0039**	**0.013**
**RBP4**	**1.22±0.40**	**1±0.45**	**-0.22**	**-0.37**	**-0.07**	**0.0040**	**0.013**
**APOC3**	**1.22±0.45**	**1±0.56**	**-0.22**	**-0.40**	**-0.04**	**0.015**	**0.041**
APOE	1.11±0.32	1±0.33	-0.12	-0.23	0.00	0.049	0.13
LCAT	1.10±0.31	1±0.34	-0.10	-0.21	0.01	0.079	0.18
PON3	1.08±0.29	1±0.28	-0.08	-0.18	0.01	0.095	0.20
AMBP	0.93±0.15	1±0.32	0.08	-0.02	0.17	0.11	0.22
PON1	1.08±0.30	1±0.30	-0.08	-0.19	0.02	0.12	0.22
SAA1	0.56±0.68	1±2.04	0.44	-0.16	1.04	0.15	0.26
PTGDS	0.68±0.24	1±1.54	0.32	-0.13	0.76	0.16	0.26
ITIH4	0.95±0.21	1±0.26	0.05	-0.03	0.13	0.23	0.33
APOA1	0.94±0.21	1±0.33	0.07	-0.05	0.20	0.23	0.33
SERPINA1	0.90±0.23	1±0.58	0.10	-0.07	0.28	0.23	0.33
HPR	1.10±0.64	1±0.45	-0.10	-0.27	0.07	0.27	0.36
B2M	0.81±0.31	1±1.35	0.19	-0.20	0.59	0.34	0.43
PLG	1.04±0.25	1±0.24	-0.04	-0.12	0.04	0.35	0.43
CETP	0.92±0.49	1±0.75	0.08	-0.15	0.32	0.48	0.56
APOC1	1.06±0.42	1±0.54	-0.06	-0.25	0.14	0.55	0.62
APOM	0.99±0.24	1±0.28	0.01	-0.08	0.10	0.80	0.86
LPA	1.02±1.14	1±1.28	-0.02	-0.43	0.40	0.94	0.95
APOA2	1.00±0.22	1±0.36	0.00	-0.11	0.11	0.95	0.95

The number of subjects was 181, including 47 healthy control subjects and 134 T1DM subjects in the cohort group without CVD events. Following digestion of reduced and alkylated serum proteins with trypsin, digested peptides were analyzed by isotope dilution targeted MS/MS with parallel reaction monitoring (PRM). The level of each serum protein from 134 cohort subjects without CVD events was defined as 1.00. Data are means ± SDs for protein levels (arbitrary unit). Unadjusted difference, 95% confidence interval (CI), and P-values were from unadjusted linear regression, in which the protein level was used as dependent variable and T1DM status was used as an independent variable. The unadjusted differences were from the unstandardized coefficients (B). Proteins significantly associated with diabetic status after controlling the Benjamini-Hochberg false discovery rate at 5% (FDR adjusted *p*-values or Q-values < 0.05) are shown in bold.

Supplemental Table 3. Unadjusted Hazard Ratios of Serum Proteins Associating with Incident CVD in T1DM Subjects in CACTI.

Protein	Level (mean±SD) (Arb. Unit)		Unadjusted HR	95% CI		*P*-value	Q-value
	T1DM Cohort (N=145)	T1DM CAD Cases (N=47)		Lower	Upper		
**AMBP**	**1.01±0.32**	**1.23±0.40**	**2.21**	**1.51**	**3.23**	**<0.0001**	**0.00057**
**APOA4**	**1.03±0.42**	**1.34±0.59**	**1.93**	**1.42**	**2.64**	**<0.0001**	**0.00057**
**APOC3**	**1.04±0.62**	**1.33±0.66**	**1.83**	**1.40**	**2.40**	**<0.0001**	**0.00072**
**RBP4**	**1.01±0.46**	**1.36±0.65**	**1.98**	**1.40**	**2.80**	**0.00010**	**0.00072**
**B2M**	**1.01±1.31**	**1.52±1.49**	**1.75**	**1.27**	**2.41**	**0.00060**	**0.0034**
**APOC2**	**1.03±0.62**	**1.26±0.67**	**1.60**	**1.19**	**2.14**	**0.0017**	**0.0068**
**ITIH4**	**1.01±0.26**	**1.12±0.26**	**1.57**	**1.19**	**2.09**	**0.0017**	**0.0068**
**PTGDS**	**1.01±1.49**	**1.57±1.86**	**1.66**	**1.20**	**2.30**	**0.0022**	**0.0077**
**A2M**	**1.01±0.40**	**1.17±0.45**	**1.60**	**1.17**	**2.19**	**0.0030**	**0.0093**
**APOB**	**1.02±0.36**	**1.15±0.35**	**1.49**	**1.13**	**1.97**	**0.0043**	**0.012**
**APOC1**	**1.01±0.54**	**1.19±0.53**	**1.45**	**1.06**	**1.98**	**0.010**	**0.026**
**PLTP**	**1.01±0.35**	**1.13±0.39**	**1.43**	**1.03**	**2.00**	**0.020**	**0.047**
APOA2	1.01±0.36	1.10±0.27	1.42	1.09	1.85	0.023	0.050
APOA1	1.01±0.33	1.07±0.21	1.32	1.04	1.67	0.034	0.068
SAA1	1.00±1.98	1.05±1.09	1.32	0.999	1.74	0.051	0.094
APOM	1.00±0.29	1.09±0.29	1.36	0.99	1.89	0.061	0.11
APOE	1.02±0.34	1.12±0.42	1.35	0.97	1.89	0.073	0.12
LCAT	1.01±0.34	1.09±0.35	1.32	0.97	1.80	0.077	0.12
APOC4	1.03±0.86	1.10±0.87	1.21	0.93	1.57	0.15	0.22
APOL1	1.00±0.32	1.07±0.37	1.19	0.85	1.66	0.31	0.40
HPR	1.00±0.45	1.09±0.51	1.19	0.83	1.71	0.34	0.40
PLG	1.00±0.25	1.05±0.27	1.19	0.84	1.68	0.34	0.40
PON3	1.00±0.28	1.05±0.29	1.18	0.85	1.63	0.32	0.40
SERPINA1	1.01±0.57	1.07±0.49	1.20	0.85	1.68	0.30	0.40
CETP	1.02±0.74	1.07±0.46	1.16	0.794	1.70	0.44	0.49
PON1	1.00±0.31	1.04±0.29	1.13	0.83	1.53	0.45	0.49
IGF2	0.99±0.32	1.06±0.38	1.17	0.70	1.95	0.55	0.57
LPA	0.98±1.25	1.16±1.40	1.03	0.70	1.50	0.89	0.89

The total number of subjects included was 181. There were 145 subjects in the T1DM cohort group and a total of 47 T1DM subjects with CVD events in CACTI (including 11 CVD subjects in the cohort group). Following digestion of reduced and alkylated serum proteins with trypsin, digested peptides were analyzed by isotope dilution targeted MS/MS with parallel reaction monitoring (PRM). The level of each serum protein from 134 cohort subjects without CVD events was defined as an arbitrary unit of 1.00. Levels are means ± SDs. Unadjusted hazard ratios (HR), 95% confidence intervals (CI) and p values of serum proteins are calculated by Cox proportional hazards models (47 subjects with events and 181 total subjects) using a case-cohort design and the "PROC PHREG" function in SAS with weighting (see methods section for details). In order to improve the model fitting, hazard ratios for CAD events are per SD increase in log transformed levels of serum proteins. Proteins significantly associated with incident CVD after controlling the Benjamini-Hochberg false discovery rate at 5% (FDR adjusted *p*-values or Q-values < 0.05) are shown in bold. See Supplemental Table 1 for protein names.
